# High-Fructose High-Fat Diet Renders the Retina More Susceptible to Blue Light Photodamage in Mice

**DOI:** 10.3390/antiox14080898

**Published:** 2025-07-22

**Authors:** Meng-Wei Kao, Wan-Ju Yeh, Hsin-Yi Yang, Chi-Hao Wu

**Affiliations:** 1 Graduate Program of Nutrition Science, School of Life Science, National Taiwan Normal University, Taipei 11677, Taiwan; kevin.dietitian@gmail.com (M.-W.K.); wandayeh@ntnu.edu.tw (W.-J.Y.); 2 Department of Nutritional Science, Fu Jen Catholic University, New Taipei City 24205, Taiwan; 144542@mail.fju.edu.tw

**Keywords:** advanced glycation end product, blue light, high-fructose high-fat diet, oxidative stress, photochemical damage, RAGE, retina

## Abstract

Retinal degeneration is associated with dietary factors and environmental light exposure. This study investigated the effects of a high-fructose high-fat (HFHF) diet on susceptibility to blue light (BL)-induced retinal damage. Male ICR mice were randomized into three groups: control, BL alone, and BL plus HFHF diet (BL + HFHF). The BL + HFHF group consumed the HFHF diet for 40 weeks, followed by 8 weeks of low-intensity BL exposure (465 nm, 37.7 lux, 0.8 μW/cm^2^) for 6 h daily. The BL group underwent the same BL exposure while kept on a standard diet. Histopathological analysis showed that, under BL exposure, the HFHF diet significantly reduced the number of photoreceptor nuclei and the thickness of the outer nuclear layer and inner/outer segments compared to the BL group (*p* < 0.05). While BL exposure alone caused oxidative DNA damage, rhodopsin loss, and Müller cell activation, the combination with an HFHF diet significantly amplified the oxidative DNA damage and Müller cell activation. Moreover, the HFHF diet increased blood–retinal barrier permeability and triggered apoptosis under BL exposure. Mechanistically, the BL + HFHF group exhibited increased retinal advanced glycated end product (AGE) deposition, accompanied by the activation of the receptor for AGE (RAGE), NFκB, and the NLRP3 inflammasome-dependent IL-1β pathway. In conclusion, this study underscores that unhealthy dietary factors, particularly those high in fructose and fat, may intensify the hazard of BL and adversely impact visual health.

## 1. Introduction

Light-emitting diodes (LEDs) generally emit relatively high levels of blue light (BL) and now have replaced conventional incandescent light bulbs in indoor lighting and electronic equipment. According to ElectronicsHub-2023, global screen time averages 6 h and 37 min daily per individual [1], which means that after deducting sleep time, each person spends approximately 40% of their waking hours exposed to BL. During the COVID-19 pandemic, online learning and work-from-home setups have indirectly increased overall BL exposure [2].

BL comprises high-energy photons with a wavelength of between 400 and 500 nm, enabling it to penetrate the eye and reach the retina. When BL photons hit the retina, they are absorbed by flavins and cytochromes within mitochondria’s electron transport chain in cells, which stimulates the overproduction of reactive oxygen species (ROS) [3]. As the retina is a tissue rich in polyunsaturated fatty acids and light-sensitive substances, exposure to BL could lead to an accumulation of oxidization products such as malonaldehyde (MDA), nitrotyrosine, and 8-hydroxy-2-deoxyguanosine (8-OHdG), destroying the integrity of the blood–retinal barrier (BRB) [4] and leading to apoptosis, necrosis, and pyroptosis of photoreceptor and retinal pigment epithelium (RPE) cells [5]. Photoreceptor and RPE cells are the primary targets of BL-induced retinal damage [6]. Clinical studies have shown that individuals who use digital devices for more than 8 h per day have declined photoreceptor cell function [7]. Experimental rats exposed to BL had reduced a and b wave amplitudes on electroretinography (ERG) and fewer nuclei in the outer nuclear layer (ONL) of the retina [8].

Recent studies have established a strong link between diet and eye health, identifying diets high in sugar and fat as significant risk factors [9,10]. Specifically, unhealthy dietary patterns, such as the Western diet—characterized by high intake of red and processed meats, high-fat dairy, french fries, and refined grains—are implicated in the development and progression of late-stage age-related macular degeneration (AMD) [11]. Long-term consumption of high-fructose high-fat (HFHF) diets can induce systemic metabolic disorders, including obesity, insulin resistance, and hyperlipidemia [12]. A key pathway linking these diets to ocular pathology is the promotion of advanced glycation end product (AGE) formation [13,14], which may be exacerbated by a high dietary glycemic index [15]. The accumulation of AGE, a recognized etiological factor in diabetic retinopathy [16,17], triggers a harmful inflammatory cascade. When AGEs engage with their receptor (RAGE), they stimulate the generation of ROS and pro-inflammatory cytokines via the activation of the NFκB [17,18,19] and NLRP3 inflammasome signaling pathways [13]. Conversely, specific dietary choices could offer significant protection. A meta-analysis of 18 high-quality studies concluded that a diet rich in carotenoid-containing vegetables and omega-3 fatty acid-rich fish is beneficial for individuals at risk of AMD. The same analysis reinforced that limiting the intake of animal fats and red/processed meats may reduce the risk of disease progression [20]. These protective principles are embodied in the Mediterranean diet. Emerging evidence now recognizes adherence to this dietary pattern as a potent intervention for preventing and ameliorating not only AMD but also other major ophthalmologic diseases, including diabetic retinopathy and glaucoma [21].

Taken together, the increasing prevalence of BL exposure, coupled with the global shift towards Westernized dietary habits, presents a growing concern for ocular health. However, the potential synergistic effects of these two lifestyle factors on retinal integrity remain poorly understood. This study, therefore, investigates how a long-term HFHF diet modulates photochemical damage in the mouse retina under environmental BL conditions. The findings aim to elucidate the interplay between diet and light exposure, thereby providing a scientific rationale for developing dietary interventions to protect against BL-induced retinal injury and preserve visual function.

## 2. Materials and Methods

### 2.1. Chemicals

Formaldehyde solution (10% *w*/*v* in aqueous phosphate buffer, H121-08) was purchased from Avantor (Radnor Township, PA, USA). Micromount (mounting medium, 3801731) was purchased from Leica (Wetzlar, Germany). The antibodies used in this study are listed in Table A1. All other chemicals and reagents were purchased from Sigma Chemical (St. Louis, MO, USA). All chemicals and solvents used in this study were of analytical grade, with a purity exceeding 99%.

### 2.2. Animals

Nine-week-old male ICR mice (n = 24) weighing 25–30 g were purchased from BioLASCO Taiwan Co., Ltd. (Yilan, Taiwan). Mice were maintained in the following specific pathogen-free standard housing conditions: temperature-controlled room (22 ± 2 °C), 50% ± 10% humidity, and a 12 h–12 h light–dark cycle (0600–1800) with food and water ad libitum. All animal experiments were conducted following the Guide for the Care and Use of Laboratory Animals (National Academy of Sciences, Taipei, Taiwan) and approved by the Institutional Animal Care and Use Committee of National Taiwan Normal University (No. 110019).

### 2.3. Mouse Model of Long-Term HFHF Diet and BL Exposure

**Figure 1:**
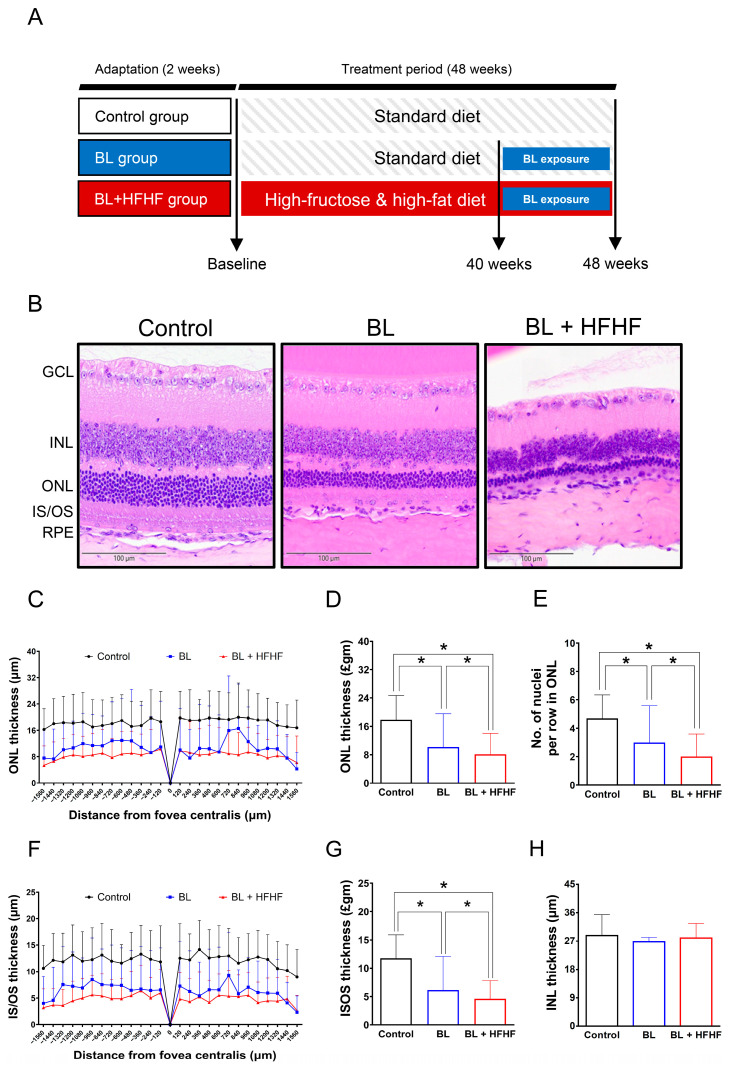
Comparative histopathological analysis of retinal morphology in ICR mice following a 40-week dietary intervention with either HFHF or standard chow diet under BL exposure. (**A**) Schematic representation of the experimental design. (**B**) Representative H&E-stained retinal cross-sections from experimental groups. (**C**,**D**) Quantitative assessment of outer nuclear layer (ONL) thickness and (**E**) photoreceptor nuclear row count in ONL. (**F**,**G**) Inner segment/outer segment (IS/OS) thickness measurements and (**H**) inner nuclear layer (INL) thickness. All morphometric analyses were performed at 120 μm intervals from the superior to the inferior edge of the retina. Data represent mean ± SD (n = 8). Intergroup differences were analyzed by two-tailed unpaired Student’s *t*-test with statistical significance denoted as * *p* < 0.05. Bars without asterisks denote non-significant differences. BL, blue light. H&E, hematoxylin and eosin. HFHF, high-fructose high-fat. ONL, outer nuclear layer. INL, inner nuclear layer. IS/OS, photoreceptor inner segment/outer segment. GCL, ganglion cell layer. RPE, retinal pigment epithelium.

Following a two-week acclimatization period, experimental animals were randomly assigned to three groups: a control group, a BL exposure group, and a group subjected to both BL exposure and an HFHF diet (BL + HFHF group). The 48-week experimental protocol was divided into two distinct phases. During the initial 40-week phase, the control and BL groups were kept on a standard chow diet, while the BL + HFHF group was fed the HFHF diet. In the subsequent 8-week phase, both the BL group and the BL + HFHF group were exposed to 465 nm BL LED light (Philips, Amsterdam, The Netherlands) at an intensity of 0.8 μW/cm^2^ (37.7 lux) for 6 h daily. Throughout this exposure period, the BL + HFHF group continued on the HFHF diet, whereas the BL group underwent identical BL exposure while remaining on the standard diet (Figure 1A). The HFHF diet provided 60% of its calories from fat (Research Diet D12492, Research Diet Inc., New Brunswick, NJ, USA) and was supplemented with 15% *w*/*v* fructose in the drinking water (102186, Irvine, CA, USA).

### 2.4. Intraperitoneal Glucose Tolerance Test (IPGTT) and Biochemical Blood Analysis

Blood glucose levels were measured using the Abbott Freestyle Optium Neo (Alameda, CA, USA). After a 12 h fast, the mice received an intraperitoneal injection of a sterilized glucose solution at a dose of 2 g/kg body weight. Blood glucose levels were measured at 0, 15, 30, 60, 90, and 120 min following the injection, and the area under the curve (AUC) was calculated to evaluate glucose tolerance. Serum triglyceride levels were analyzed by Le Zen Reference Lab (Taipei, Taiwan).

### 2.5. Procedures for Eye Fixation and Histopathological Analysis

**Figure A1:**
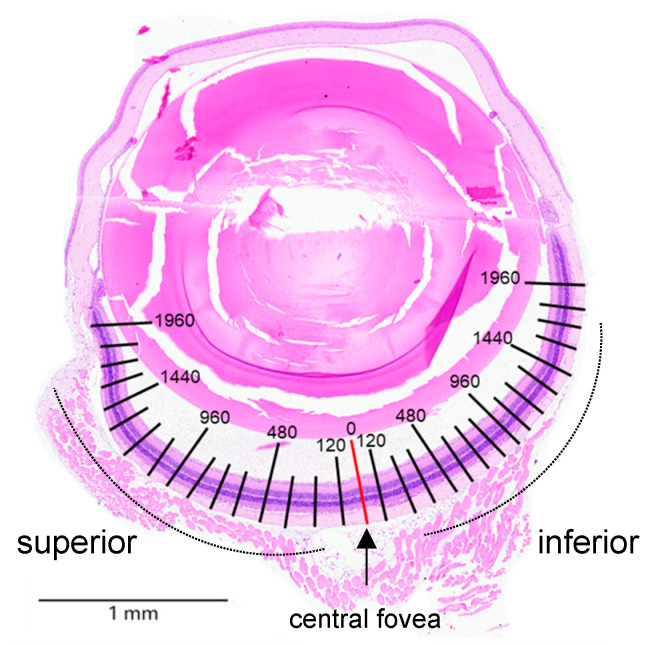
Morphometric quantification of the thicknesses of the ONL, inner/outer segments (IS/OSs), and inner nuclear layer (INL), as well as the number of nuclei in the ONL, were measured at each 120 μm intervals from the superior to inferior edge of the retina. Hematoxylin and eosin staining was performed to assess histopathological changes in the retinal tissue.

ICR mice were anesthetized with 4% isoflurane and subsequently euthanized. The eyeballs were carefully removed and fixed overnight in Davidson solution (PanReac Ap-pliChem, Darmstadt, Germany). Tissues were then dehydrated through a graded ethanol series (70%, 90%, and 100%), embedded in paraffin, and sectioned. Hematoxylin and eosin (H&E) staining was performed to assess histopathological changes in the retinal tissue. Morphometric quantification of the thicknesses of ONL, inner/outer segments (IS/OSs), and inner nuclear layer (INL), as well as the number of nuclei in the ONL, were measured using ViewPoint Light software 1.0.0.9628v (Precipoint GmbH, Munich, Germany) at 120 μm intervals from the superior to the inferior edge of the retina (Figure A1).

### 2.6. Immunofluorescence Staining

Paraffin-embedded retinal sections were prepared for immunofluorescence staining. Sections were first incubated at 57 °C for 1 h and then immersed in xylene before being rehydrated through a graded series of ethanol solutions (100%, 95%, and 70%). Antigen retrieval was performed for 10 min using an antigen retrieval pot (Bio SB, Inc., Santa Barbara, CA, USA). Sections were then washed three times with Tris-buffered saline with Tween 20 and blocked with 1% bovine serum albumin for 30 min. Sections were incubated with the indicated primary antibody at 4 °C for 14 h followed by secondary antibody incubation at 20 °C for 1 h. Nuclei were counterstained with DAPI solution (GeneTex, Irvine, CA, USA), and the slides were mounted and sealed. Images were acquired using the EVOS^®^ FL Auto Imaging System (Thermo Fisher Scientific, Waltham, MA, USA) at 20× magnification. Fluorescence intensity was quantified using ImageJ software 1.54g.

### 2.7. Serum MDA and Fluorescent AGE Levels

Serum MDA levels and AGE-associated fluorescence were measured using previously established methods [22]. For AGE analysis, 50 μL of serum was incubated with 250 μL of a reducing buffer (consisting of 0.2 M sodium borate buffer and 1 M sodium borohydride in 0.01 M NaOH) for 2 h. The reaction mixture was then centrifuged at 10,000× *g* for 2 min, and the supernatant was collected. Lipids were extracted from the supernatant using a methanol–chloroform solution. Subsequently, 250 μL of 6 N HCl was added, and the mixture was heated at 110 °C for 24 h in a dry bath. The final supernatant was filtered through a 0.22 μm filter. Individual fluorescent AGEs were determined by scanning for specific AGE fluorescence at the following wavelengths: crossline (Ex. 379 nm; Em. 463 nm), fluorolink (Ex. 380 nm; Em. 460 nm), 2-(2-furoyl)-4(5)-(2-furanyl)-1H-imidazole (FFI; Ex. 380 nm; Em. 440 nm), lysylpyropyridine (Ex. 370 nm; Em. 448 nm), and vesperlysine A and B (Ex. 366 nm; Em. 442 nm).

### 2.8. TUNEL Assay

The TUNEL assay was conducted using the One-step TUNEL In Situ Apoptosis Kit (Elab Fluor^®^ 488, DAPI; Cat No. E-CK-A321, Elabscience^®^, Houston, TX, USA) on 4 μm paraffin sections. After deparaffinization and rehydration, sections were treated with protease K (37 °C, 20 min) and DNase I buffer (25 °C, 5 min), with PBS washes between steps. The sections were subsequently stained with the labeling working solution and incubated for 1 h at 37 °C in the dark. Fluorescence was measured using an EVOS^®^ FL Auto Imaging System.

### 2.9. Statistical Analysis

Experimental data were expressed as mean ± SD for n = 8. Data analysis and plotting were conducted using Prism version 6.0 software. Comparisons between groups were performed using the unpaired *t*-test. Differences were considered statistically significant at a *p*-value of less than 0.05.

## 3. Results

### 3.1. Physiological and Biochemical Changes After 40 Weeks of HFHF Diet

**Figure A2:**
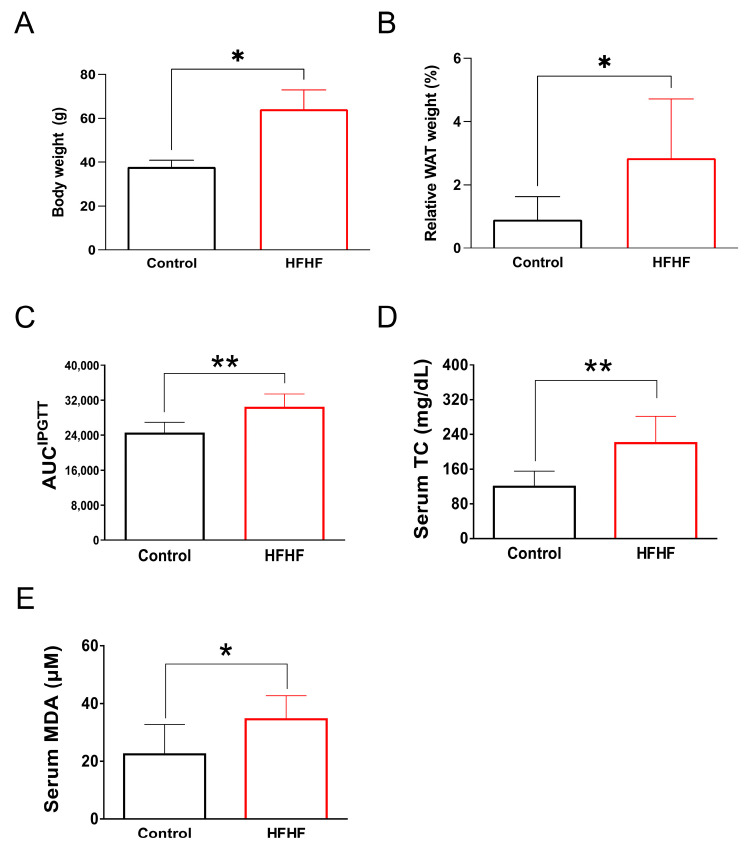
Long-term administration of an HFHF diet for 40 weeks in ICR mice induced characteristic metabolic abnormalities, as evidenced by (**A**) increased body weight, (**B**) enhanced visceral fat accumulation, (**C**) impaired glucose tolerance, and (**D**) elevated serum levels of total triglycerides and (**E**) the lipid peroxidation product MDA. Values are expressed as mean ± SD (n = 8). Statistical comparisons between groups were performed using unpaired Student’s *t*-test. * *p* < 0.05; ** *p* < 0.01. AUC, area under the curve; HFHF, high-fructose high-fat; IPGTT, intraperitoneal glucose tolerance test; MDA, malondialdehyde; WAT, white adipose tissue.

This study examined the long-term effects of an HFHF diet on systemic metabolic parameters and retinal health in mice. After 40 weeks of HFHF diet administration, the mice exhibited notable metabolic abnormalities. Quantitative analyses revealed statistically significant increases in body weight and visceral adiposity index (*p* < 0.05) compared to control groups. Metabolic profiling demonstrated impaired glucose tolerance during intraperitoneal glucose tolerance tests (AUC increase 23.9%, *p* < 0.01), accompanied by elevated serum triglycerides (1.9-fold increase, *p* < 0.01) and MDA levels (1.5-fold increase, *p* < 0.05), indicating systemic lipid peroxidation (Figure A2). These findings confirm the detrimental metabolic effects of prolonged HFHF dietary intake.

### 3.2. Potentiation of BL-Induced Photoreceptor Degeneration by HFHF Diet

Following the characterization of metabolic disturbances in HFHF-fed mice, animals were exposed to low-intensity BL (6 h/day) for 8 weeks while maintaining the HFHF diet. The experimental design is schematically illustrated in Figure 1A. Histological examination of H&E-stained retinal cross-sections from different experimental groups is shown in Figure 1B. Morphometric measurements of retinal-layer thickness, including the ONL, photoreceptor IS/OSs, and INL, as well as quantification of nuclei within the ONL, were conducted at 120 μm intervals, as indicated in Materials and Methods. Quantitative analysis demonstrated that BL exposure alone led to a significant reduction in photoreceptor integrity, manifesting as a 36% decrease in the number of ONL nuclei, along with 38% and 47% decreases in ONL and IS/OS thickness, respectively, compared to control (Figure 1C–G; *p* < 0.05). Notably, mice subjected to the combined BL + HFHF treatment exhibited more serve retinal damage than those exposed to BL alone. The BL + HFHF group exhibited greater reductions in ONL nuclei count as well as ONL and IS/OS thickness compared to the BL-only group (Figure 1C–G, *p* < 0.05). In contrast, no statistically significant differences in INL thickness were observed among all groups (Figure 1H, *p* > 0.05), indicating that the observed retinal damage was predominantly localized to the photoreceptor layers.

### 3.3. HFHF Diet Potentiates Oxidative Stress, Triggers Apoptosis, and Promotes the Activation of Müller Cells Under BL Exposure

**Figure 2:**
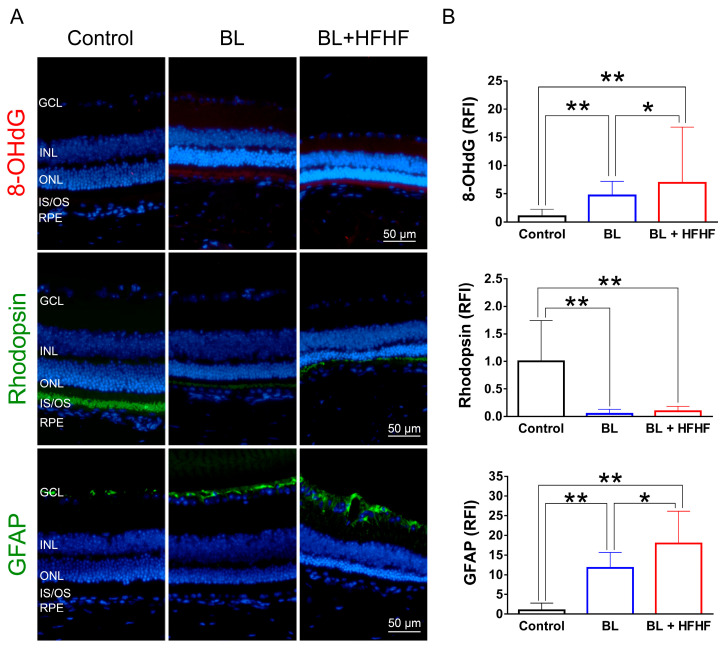
HFHF diet exacerbates BL-induced oxidative stress and downregulates rhodopsin and GFAP expression. ICR mice were fed an HFHF diet or standard chow for 40 weeks, followed by exposure to BL for 8 weeks. (**A**) Representative immunofluorescence images showing retinal expression patterns of 8-OHdG (oxidative damage marker), rhodopsin (photoreceptor marker), and GFAP (glial activation marker). (**B**) Quantitative analysis of relative fluorescence intensity for each marker. Data represent mean ± SD (n = 8). Intergroup differences were assessed by two-tailed unpaired Student’s *t*-test (* *p* < 0.05, ** *p* < 0.01, bars without asterisks denote non-significant differences). BL, blue light. HFHF, high-fructose high-fat. ONL, outer nuclear layer. INL, inner nuclear layer. IS/OS, photoreceptor inner segment/outer segment. GCL, ganglion cell layer. GFAP, glial fibrillary acidic protein. RFI, relative fluorescence intensity. RPE, retinal pigment epithelium.

**Figure 3:**
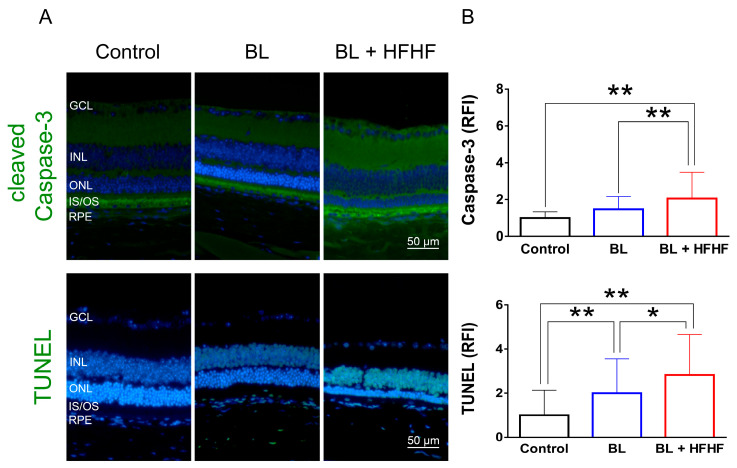
HFHF diet exacerbates BL-induced retinal apoptosis. Retinal apoptosis was assessed in tissue sections using cleaved caspase-3 staining and the TUNEL assay. Mice were fed an HFHF diet or standard chow for 40 weeks, followed by exposure to BL for 8 weeks. (**A**) Representative immunofluorescence images showing retinal expression patterns of cleaved caspase-3 and TUNEL-positive cells. (**B**) Quantitative analysis of relative fluorescence intensity for each marker. Data represent mean ± SD (n = 8). Intergroup differences were assessed by two-tailed unpaired Student’s *t*-test (* *p* < 0.05, ** *p* < 0.01, bars without asterisks denote non-significant differences). BL, blue light. HFHF, high-fructose high-fat. ONL, outer nuclear layer. INL, inner nuclear layer. IS/OS, photoreceptor inner segment/outer segment. GCL, ganglion cell layer. RFI, relative fluorescence intensity. RPE, retinal pigment epithelium.

To assess the extent of oxidative damage induced by BL and the HFHF diet, we measured 8-OHdG levels, a well-established marker of DNA oxidative damage. As shown in Figure 2 (upper panel), retinal 8-OHdG levels were significantly elevated in the BL group compared to the control group (4.7-fold, *p* < 0.01). This increase was further exacerbated in the BL + HFHF group, showing a 1.4-fold rise compared to the BL group (*p* < 0.05) and an overall seven-fold increase compared to the control group (*p* < 0.01). We next examined the effect of BL exposure on rhodopsin expression, the predominant protein in the OS discs of photoreceptor cells. BL exposure led to a substantial reduction in rhodopsin levels, with no significant difference observed between the BL-only and BL + HFHF groups (Figure 2, middle panel; *p* > 0.05). Consistent with these findings, we assessed GFAP expression in Müller cells, a marker of retinal damage [8]. BL exposure significantly activated Müller cells by increased GFAP protein expression. Notably, the BL + HFHF group exhibited approximately 1.5-fold greater GFAP expression than the BL group (Figure 2, bottom panel; *p* < 0.05). As shown in Figure 3, analysis of cleaved caspase-3 protein expression and TUNEL staining revealed that mice exposed to low-intensity BL alone for eight weeks exhibited apoptosis, whereas the combination of BL and the HFHF diet resulted in significantly exacerbated retinal cell apoptosis compared to the BL-only group. These findings suggest that an HFHF diet potentiates the damaging effects of BL on the retina.

### 3.4. HFHF Diet Disrupts BRB Integrity and Promotes Lipofuscin Accumulation Under BL Exposure

**Figure 4:**
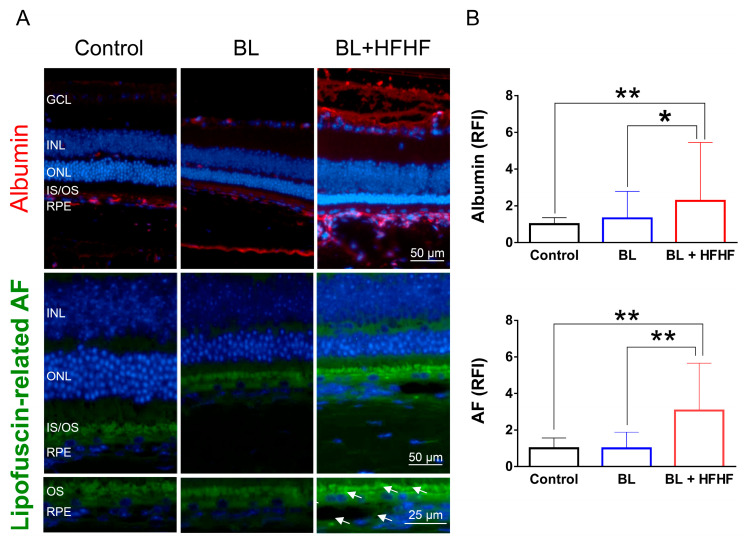
HFHF diet induces BRB leakage and promotes lipofuscin accumulation under BL exposure. The integrity of the BRB was evaluated in retinal sections using albumin staining, while lipofuscin accumulation was assessed by measuring relative autofluorescence. Mice were fed a HFHF diet or standard chow for 40 weeks, followed by exposure to BL for 8 weeks. (**A**) Representative immunofluorescence images showing albumin leakage and lipofuscin-related autofluorescence in the retina. (**B**) Quantitative analysis of relative fluorescence intensity for each marker. Data represent mean ± SD (n = 8). Intergroup differences were assessed by two-tailed unpaired Student’s *t*-test (* *p* < 0.05, ** *p* < 0.01, bars without asterisks denote non-significant differences). AF, autofluorescent. BL, blue light. BRB, blood–retinal barrier. HFHF, high-fructose high-fat. ONL, outer nuclear layer. INL, inner nuclear layer. IS/OS, photoreceptor inner segment/outer segment. GCL, ganglion cell layer. RFI, relative fluorescence intensity. RPE, retinal pigment epithelium.

The retina is protected by an inner and outer BRB, which prevents the entry of macromolecules and harmful substances. Leakage of albumin into the retina indicates a disruption of this barrier [4]. Figure 4 shows that low-intensity BL exposure did not compromise the integrity of BRB. However, mice subjected to both the HFHF diet and BL exhibited a significant increase in albumin-associated fluorescent intensity in the retina, with levels rising by 2.2 times (*p* < 0.05), suggesting BRB disruption. Similarly, while eight weeks of BL exposure did not cause significant lipofuscin accumulation in the retina, the combination of the HFHF diet and BL exposure led to a marked increase in autofluorescence compared to both the BL-only group and the control group (*p* < 0.01). These autofluorescent spots were found to cluster and accumulate specifically within the OS and RPE layers of the BL + HFHF group (Figure 4A, bottom panel).

### 3.5. HFHF Diet Induces the Formation of AGEs and Triggers the Activation of RAGE and NFκB in the Retina

**Figure 5:**
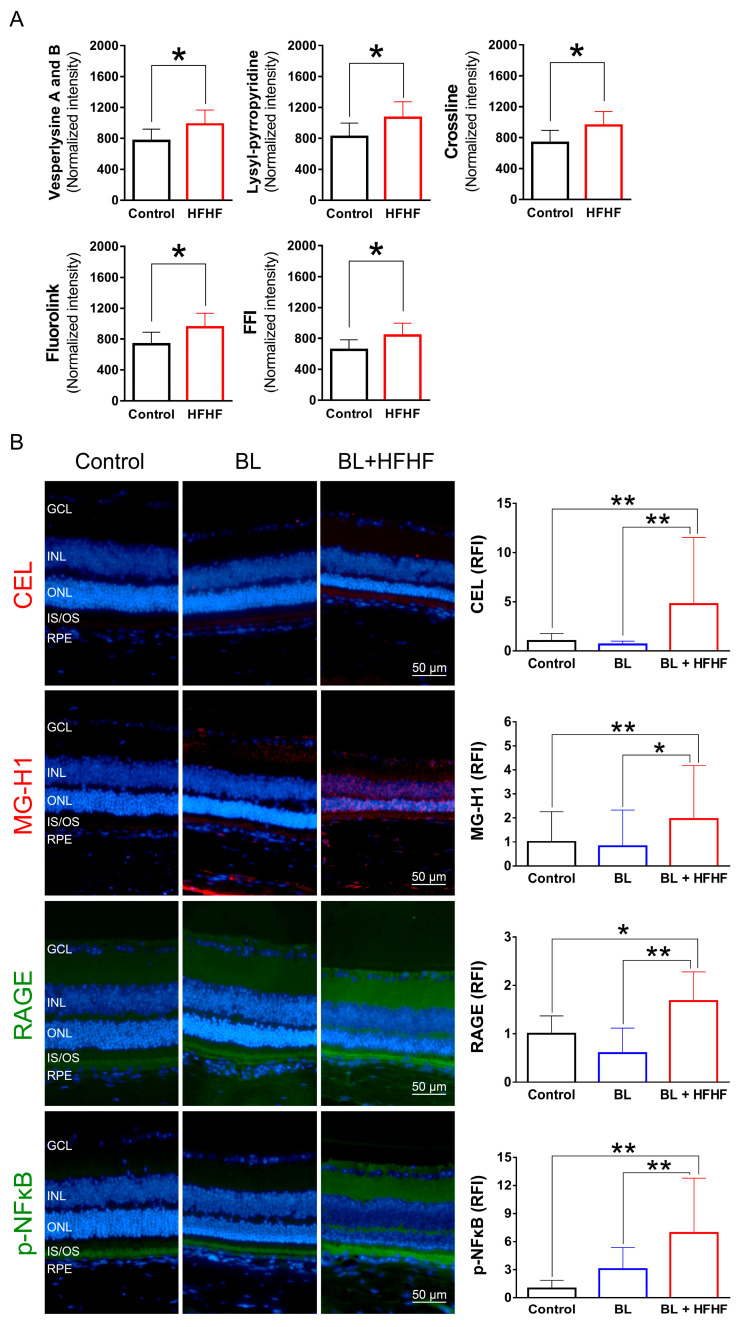
HFHF diet promotes AGE formation and activates RAGE and NFκB in the retina. (**A**) Serum AGE fluorescence intensity. Mice were fed an HFHF diet or standard chow for 40 weeks. Fluorescent AGE was quantified at specific excitation/emission wavelengths: crossline (379 nm/463 nm), fluorolink (380 nm/460 nm), FFI (380 nm/440 nm), lysylpyropyridine (370 nm/448 nm), and vesperlysine A/B (366 nm/442 nm). (**B**) Immunofluorescence analysis of retinal AGE (CEL, MG-H1), RAGE, and phosphorylated NFκB. Data represent mean ± SD (n = 8). Intergroup differences were assessed by two-tailed unpaired Student’s *t*-test (* *p* < 0.05, ** *p* < 0.01, bars without asterisks denote non-significant differences). AGE, advanced glycated end products. BL, blue light. FFI, 2-furoyl-4 (5)-(2-furanyl)-1H-imidazole. HFHF, high-fructose high-fat. ONL, outer nuclear layer. INL, inner nuclear layer. IS/OS, photoreceptor inner segment/outer segment. GCL, ganglion cell layer. RAGE, receptor for AGE. RFI, relative fluorescence intensity. RPE, retinal pigment epithelium.

To investigate the effects of an HFHF diet on AGE formation and potential retinal damage, we analyzed serum AGE-associated fluorescence using three-dimensional fluorescence spectroscopy. As shown in Figure 5A, mice fed an HFHF diet exhibited significantly elevated levels of fluorescent AGE markers, including crossline, fluorolink, FFI, lyylpyrropyridine, and vesperlysine A and B, compared to those fed a standard chow diet (*p* < 0.05). To further elucidate the effects of the HFHF diet on retinal tissue, we examined retinal AGE levels using immunofluorescence staining. Notably, BL exposure alone did not induce retinal AGE formation or RAGE expression. However, mice subjected to both the HFHF diet and BL led to a significant increase in the expression of Nε-(carboxyethyl)lysine (CEL) and methylglyoxal-derived hydroimidazolone (MG-H1), as well as RAGE activation (Figure 5B). The AGE–RAGE interaction is known to initiate downstream signaling cascades, ultimately evoking the phosphorylation of NFκB [18]. Analysis of p-NFκB levels revealed no statistically significant difference between the control and BL groups (*p* > 0.05). In contrast, the BL + HFHF group exhibited a significant increase in p-NFκB levels compared to both the control group and the BL groups (*p* < 0.01). These findings reveal that long-term consumption of an HFHF diet may promote AGE/RAGE-mediated NFκB activation, potentially exacerbating retinal damage under BL exposure.

### 3.6. HFHF Diet Triggers NLRP3 Inflammasome Activation Under BL Exposure

**Figure 6:**
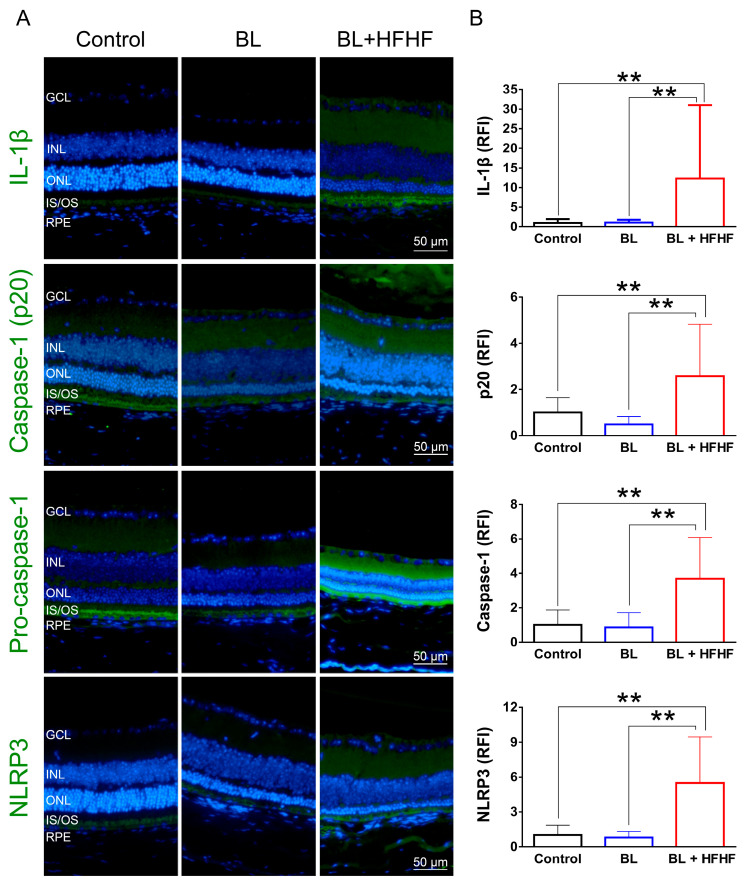
Activation of the NLRP3 inflammasome-IL-1β pathway in the retina by HFHF diet under BL exposure. The expression of IL-1β and NLRP3 inflammasome components was examined by immunofluorescence staining in mice fed an HFHF diet or standard chow for 40 weeks, followed by 8 weeks of BL exposure. (**A**) Representative immunofluorescence images showing retinal expression patterns of IL-1β and NLRP3 inflammasome pathway. (**B**) Quantitative analysis of relative fluorescence intensity for each marker. Data represent mean ± SD (n = 8). Intergroup differences were assessed by two-tailed unpaired Student’s *t*-test (** *p* < 0.01, bars without asterisks denote nonsignificant differences). BL, blue light. HFHF, high-fructose high-fat. ONL, outer nuclear layer. INL, inner nuclear layer. IS/OS, photoreceptor inner segment/outer segment. GCL, ganglion cell layer. RFI, relative fluorescence intensity. RPE, retinal pigment epithelium.

As shown in Figure 6, low-intensity BL exposure did not increase the expression of proinflammatory cytokine IL-1β in the retina. However, the BL + HFHF group exhibited a significant increase in IL-1β expression compared to both the control group and the BL group (*p* < 0.01). Mechanistically, this group showed elevated expression levels of NLRP3, pro-caspase-1, and caspase-1—key components of the inflammasome pathway. These findings suggest that the HFHF diet may promote retinal inflammation, particularly under BL exposure.

## 4. Discussion

The retina is particularly vulnerable to photodamage due to its high content of polyunsaturated fatty acids, with photoreceptor OSs containing approximately 60% docosahexaenoic acid [23]. Consequently, ONL and IS/OSs are very susceptible sites for attack by ROS. BL irradiation has been shown to stimulate the overproduction of superoxide anions and hydrogen peroxide, leading to lipid peroxidation and DNA damage and ultimately resulting in retinal cell death [24]. Approximately 40–60% of digital screen-emitted BL is estimated to penetrate the eye and reach the retina [25]. Although direct clinical evidence linking BL exposure to human retinal damage is lacking, animal studies have shown that BL exposure can cause morphological abnormalities in retinal tissue, including thinning of retinal layers, distortion of the boundaries, shrinkage of photoreceptor nuclei, and a decreased number of photoreceptor nuclei [26,27]. These pathological injuries are often accompanied by increased GFAP expression in Müller cells, a sensitive indicator of retinal stress in response to BL exposure [8].

Studies on BL photodamage typically focus on wavelengths between 420 and 470 nm. Notably, 460 nm BL causes more severe oxidative damage and functional changes in rats compared to 530 nm (green) and 620 nm (red) light [26]. Interestingly, Tosini et al. [28] found that exposing anesthetized rats to 470–480 nm BL for four weeks (4 h/d) had no damaging effect on their photoreceptor cells. To simulate daily environmental conditions, we utilized BL LEDs with a wavelength of 465 nm and set the light intensity to 37.7 lux (0.8 μW/cm^2^), which is below the typical workplace lighting of less than 1000 lux [29] and considered low intensity [7,30]. We also set the total cumulative BL exposure to 6 h per day, based on the average global daily use of digital screens [1]. This experimental approach allowed us to investigate the impact of an HFHF diet on photodamage under conditions that closely mimic real-world exposure patterns. Furthermore, to minimize potential interference from estrogen, male mice were specifically chosen based on evidence from animal models demonstrating that 17β-estradiol acts as an antioxidant, providing protection against light-induced retinal degeneration. This protective effect is thought to occur through a reduction in apoptosis and the enhancement of autophagy [31].

The main question addressed by this study is whether an HFHF diet affects retinal photodamage in mice. We hypothesize that long-term HFHF diet consumption leads to metabolic abnormalities that may exacerbate BL-induced retinal damage through enhanced oxidative stress and inflammatory responses. The underlying mechanism in-volves the interaction between AGEs and their receptor RAGE, linking the unhealthy dietary factors and environmental BL exposure to ocular health. Our findings demonstrate that the HFHF diet significantly exacerbates the toxic effects of BL on photoreceptor cells and suggest the synergistic effects of metabolic stress and photic injury on retinal health. We observed markedly higher levels of oxidative stress, inflammation, and apoptotic responses in the retinal tissues of the BL + HFHF group compared to the BL-only group. Specifically, only the BL + HFHF group exhibited disruption of the BRB, accumulation of AGEs, and activation of RAGE. These observations underscore the detrimental synergistic effect of an HFHF diet and BL exposure on retinal health.

A diet high in fat and refined sugar, often categorized as a Westernized diet [12], is known to disrupt blood glucose and lipid regulation, potentially contributing to the development of diabetic retinopathy [32]. This detrimental impact on retinal health is further supported by studies demonstrating that high-fat diets can lead to elevated levels of MDA, a marker of oxidative stress, in the retinas of rats [33]. More specifically, the HFHF diet has been shown to induce a range of negative effects on retinal structure and function. These include alterations in retinal lipid composition, an increased n-6/n-3 fatty acid ratio [34], lipid deposition in the fundus [35], and the triggering of inflammatory responses [10]. These changes are often accompanied by an attenuated ERG response, indicating compromised retinal function [10]. Chang et al. [36] further suggests that hyperglycemia and insulin resistance, often associated with high-fat diets, may be responsible for the decline in retinal ERG responses observed in mice after 12 weeks of high-fat diet consumption. In addition to the effects of high-fat intake, excessive fructose consumption has been shown to promote the formation of reactive dicarbonyl species and AGEs [37]. A significant increase in carboxymethyllysine concentration was observed in the plasma and gastrocnemius muscle of mice fed a 60% high-fructose diet [38].

AGEs have been identified as both contributing factors to and consequences of metabolic diseases in humans [13,18,19]. These glycated compounds can bind to their receptor, RAGE, on cell membranes, activating NF-κB and leading to the formation of ROS and the release of proinflammatory cytokines [19]. The importance of AGEs in metabolic control has been highlighted by studies showing that monitoring circulating AGE fluorescence levels can effectively mirror AGE content in tissues. Additionally, increased levels of CEL and MG-H1 have been observed in the retinas of mice fed a high-glycemic-index diet [39]. In the present study, long-term intake of the HFHF diet in mice led to increased serum AGE fluorescence and accumulation of non-fluorescent CEL and MG-H1 in retinal tissues, accompanied by activation of RAGE (Figure 4). These findings are consistent with previous research and suggest for the first time that the AGE–RAGE axis plays a crucial role in the development of retinal damage.

Lipofuscin is a yellow-brown autofluorescent product that accumulates over time in the RPE layer when RPE cells cannot metabolize it efficiently. This heterogeneous complex consists of oxidized lipids, proteins, carbohydrates, photosensitive bisretinoids, and AGEs. Its highly crosslinked structure prevents degradation by the proteasome or removal via exocytosis [40]. The accumulation of lipofuscin in the retina is associated with age-related macular degeneration-like diseases, and long-term consumption of a high-fat diet has been shown to promote lipofuscin accumulation in the mouse retina [35]. In our study, we observed that the combination of an HFHF diet and BL exposure resulted in a marked increase in autofluorescence spots located within the IS/OSs and RPE layers, suggesting that HFHF diet consumption may further enhance lipofuscin accumulation (Figure 3). Moreover, lipofuscin may act as a photosensitizer, triggering the overproduction of ROS under BL exposure [3]. This hypothesis is supported by the elevated levels of MDA and 8-OHdG observed in the BL + HFHF groups.

The BRB is essential for maintaining retinal homeostasis, and its disruption can contribute to the pathogenesis of various retinal diseases. However, oxidative stress and inflammation can disrupt the structural integrity of these tight junctions, compromising BRB function [41]. For instance, STZ-treated mice fed a high-fat and high-sugar diet exhibited reduced expression of zonula occludens-1 and occludin in retinal tissue, resulting in albumin leakage from the retina and indicating disruption of BRB integrity [42]. Interestingly, exposure to high-intensity BL has been shown to disrupt BRB integrity [4]. However, our study found that exposure of mice to low-intensity BL for eight weeks did not cause BRB damage, as assessed by retinal albumin leakage (Figure 3). This result is consistent with the findings of Chan et al. [43], suggesting that BL intensity may be a critical factor in determining the degree of BRB damage. Notably, the present study demonstrates for the first time that an HFHF diet is detrimental to BRB integrity, even in a low-intensity BL environment. This observation underscores the importance of considering both environmental and dietary factors when assessing and managing retinal health.

In addition, the results presented in Figure 5 reveal an interplay between dietary factors and BL exposure in the context of retinal inflammation. Low-intensity BL exposure did not elevate IL-1β expression in retinal tissues; however, the combination of BL and an HFHF diet resulted in a marked increase in IL-1β expression. Mechanistically, the elevated expression levels of NLRP3, pro-caspase-1, and caspase-1 in the BL + HFHF group point to the activation of the NLRP3 inflammasome pathway. This observation is consistent with previous studies, which have shown that chronic administration of a high-fat diet to mice significantly increases IL-1β expression [44]. Furthermore, the HFHF diet has been linked to increased formation of AGEs and subsequent activation of the AGE–RAGE axis [13,14]. Therefore, it is plausible that the HFHF diet, by promoting AGE formation and RAGE activation, triggers NLRP3 inflammasome assembly and activation, leading to increased IL-1β production and exacerbating BL-induced retinal damage.

## 5. Conclusions

This study shows that an HFHF diet exacerbates BL-induced retinal damage by increasing photoreceptor loss, oxidative damage, Müller cell activation, and BRB permeability while promoting apoptosis. Mechanistically, the HFHF diet enhances AGE accumulation and activates RAGE, NFκB, and NLRP3 inflammasome pathways. In conclusion, diets high in fructose and fat may intensify BL hazard and threaten visual health.

## Data Availability

Data are contained within the article.

## Abbreviations

The following abbreviations are used in this manuscript:

AGE	Advanced glycated end products
AMD	Age-related macular degeneration
AUC	Area under curve
BL	Blue light
BRB	Blood–retinal barrier
CEL	Nε-(1-carboxyethyl)lysine
CML	Nε-(carboxymethyl)lysine
ERG	Electroretinogram
FFI	2-(2-furoyl)-4(5)-(2-furanyl)-1H-imidazole
GFAP	Glial fibrillary acidic protein
H&E	Hematoxylin and eosin
HFHF	High-fructose high-fat
IL-1β	Interleukin-1 beta
INL	Inner nuclear layer
IPGTT	Intraperitoneal glucose tolerance test
IS/OS	Inner/outer segment
LED	Light-emitting diodes
MDA	Malonaldehyde
MG-H1	Nδ-(5-methyl-4-imidazolon-2-yl)-L-ornithine
NFκB	Nuclear factor kappa-light-chain-enhancer of activated B cells
Nrf2	Nuclear factor erythroid 2-related factor 2
8-OHdG	8-hydroxy-2-deoxyguanosine
ONL	Outer nuclear layer
RAGE	Receptor for advanced glycated end product
RFI	Relative fluorescence intensity
ROS	Reactive oxygen species
RPE	Retinal pigment epithelium
TNF-α	Tumor necrosis factor—alpha
TUNEL	Terminal deoxynucleotidyl transferase dUTP nick end labeling
WAT	White adipose tissue

## Appendix A

**Table A1:** Listed of antibodies used for immunofluorescence analysis in this study.

Antibody (Ab)	Company/Country	Catalog No.	Dilution
Anti-GFAP Ab	Merk/Germany	AB5804	1:1000
Anti-rhodopsin Ab (Rho 4D2)	Abcam/UK	ab9887	1:1000
Cleaved caspase-3 rabbit mAb (Asp175, 5A1E)	Cell Signaling Technology/USA	9664	1:1600
IL-1β/IL-1F2 Ab	Novus Biologicals/USA	NB600-633	1:500
TNF alpha Ab	ProteinTech/USA	60291-1-Ig	1:250
8-OHdG Ab (15A3)	Novus Biologicals/USA	NB110-96878	1:750
Anti-RAGE Ab	Abcam/UK	ab3611	1:200
Anti-Nε-(carboxyethyl) lysine (CEL) Ab	COSMO BIO/Japan	AGE-M02	1:200
Anti-AGE monoclonal Ab	TransGenic/Japan	KH001	1:200
Anti-methylglyoxal (MG-H1) Monoclonal Ab	CELL BIOLABS/USA	STA-011	1:400
Mouse albumin Ab	Bethyl/USA	A90-134A	1:800
Phospho-NF-κB p65 (Ser536) (93H1) rabbit mAb	Cell Signaling Technology/USA	3033	1:1600
Recombinant Anti-NLRP3 Ab	Abcam/UK	ab263899	1:500
Caspase-1 Ab	Affinity Biosciences/ Ohio, USA	AF5418	1:200
Cleaved caspase-1 (Asp296), p20 Ab	Affinity Biosciences/USA	AF4005	1:200
Alexa Fluor^®^ 488 goat anti-rabbit igG H&L	Abcam/UK	ab150077	1:400
Alexa Fluor^®^ 488 goat anti-mouse igG H&L	Abcam/UK	ab150113	1:800
Alexa Fluor^®^ 568	Abcam/UK	ab175473	1:400

## References

[1] Kepios Pte. Ltd. (2023). Digital 2023 Global Digital Overview.

[2] Trott M., Driscoll R., Iraldo E., Pardhan S. (2022). Changes and correlates of screen time in adults and children during the COVID-19 pandemic: A systematic review and meta-analysis. EClinicalMedicine.

[3] Yeh W.J., Chien P.T., Wen Y.T., Wu C.H. (2024). A comprehensive review of experimental models for investigating blue light-induced ocular damage: Insights into parameters, limitations, and new opportunities. Exp. Eye Res..

[4] Geiger P., Barben M., Grimm C., Samardzija M. (2015). Blue light-induced retinal lesions, intraretinal vascular leakage and edema formation in the all-cone mouse retina. Cell Death Dis..

[5] Shang Y.M., Wang G.S., Sliney D., Yang C.H., Lee L.L. (2014). White light–emitting diodes (LEDs) at domestic lighting levels and retinal injury in a rat model. Environ. Health Perspect..

[6] Tao J.X., Zhou W.C., Zhu X.G. (2019). Mitochondria as potential targets and initiators of the blue light hazard to the retina. Oxidative Med. Cell. Longev..

[7] Li H., Zhang M., Wang D., Dong G., Chen Z., Li S., Sun X., Zeng M., Liao H., Chen H. (2021). Blue light from cell phones can cause chronic retinal light injury: The evidence from a clinical observational study and a SD rat model. BioMed Res. Int..

[8] Ziółkowska N., Lewczuk B., Szyryńska N., Rawicka A., Vyniarska A. (2023). Low-intensity blue light exposure reduces melanopsin expression in intrinsically photosensitive retinal ganglion cells and damages mitochondria in retinal ganglion cells in Wistar rats. Cells.

[9] Rinninella E., Mele M.C., Merendino N., Cintoni M., Anselmi G., Caporossi A., Gasbarrini A., Minnella A.M. (2018). The Role of Diet, Micronutrients and the Gut Microbiota in Age-Related Macular Degeneration: New Perspectives from the Gut–Retina Axis. Nutrients.

[10] Clarkson-Townsend D.A., Douglass A.J., Singh A., Allen R.S., Uwaifo I.N., Pardue M.T. (2021). Impacts of high fat diet on ocular outcomes in rodent models of visual disease. Exp. Eye Res..

[11] Joyal J.S., Gantner M.L., Smith L.E.H. (2018). Retinal energy demands control vascular supply of the retina in development and disease: The role of neuronal lipid and glucose metabolism. Prog. Retin. Eye Res..

[12] Malesza I.J., Malesza M., Walkowiak J., Mussin N., Walkowiak D., Aringazina R., Bartkowiak-Wieczorek J., Mądry E. (2021). High-Fat, Western-Style Diet, Systemic Inflammation, and Gut Microbiota: A Narrative Review. Cells.

[13] Ruiz H.H., Ramasamy R., Schmidt A.M. (2020). Advanced glycation end products: Building on the concept of the “common soil” in metabolic disease. Endocrinology.

[14] Aimaretti E., Chimienti G., Rubeo C., Di Lorenzo R., Trisolini L., Bello F.D., Moradi A., Collino M., Lezza A.M.S., Aragno M. (2023). Different effects of high-fat/high-sucrose and high-fructose diets on advanced glycation end-product accumulation and on mitochondrial involvement in heart and skeletal muscle in mice. Nutrients.

[15] Chiu C.-J., Milton R.C., Gensler G., Taylor A. (2007). Association between dietary glycemic index and age-related macular degeneration in nondiabetic participants in the Age-Related Eye Disease Study. Am. J. Clin. Nutr..

[16] Varoniukaite A., Verkauskiene R., Simoniene D., Paskeviciene D., Balciuniene V. (2025). Advanced glycation end products association with diabetic retinopathy severity. Acta Ophthalmol..

[17] Zhang Y., Zhang Z., Tu C., Chen X., He R. (2025). Advanced Glycation End Products in Disease Development and Potential Interventions. Antioxidants.

[18] Lin J.A., Wu C.H., Yen G.C. (2018). Perspective of advanced glycation end products on human health. J. Agric. Food Chem..

[19] Shen C.Y., Lu C.H., Wu C.H., Li K.J., Kuo Y.M., Hsieh S.C., Yu C.L. (2020). The Development of Maillard Reaction, and Advanced Glycation End Product (AGE)-Receptor for AGE (RAGE) Signaling Inhibitors as Novel Therapeutic Strategies for Patients with AGE-Related Diseases. Molecules.

[20] Chapman N.A., Jacobs R.J., Braakhuis A.J. (2019). Role of diet and food intake in age-related macular degeneration: A systematic review. Clin. Exp. Ophthalmol..

[21] Sbai O., Torrisi F., Fabrizio F.P., Rabbeni G., Perrone L. (2024). Effect of the mediterranean diet (MeDi) on the progression of retinal disease: A narrative review. Nutrients.

[22] Khoo S.H., Wu P.R., Yeh K.T., Hsu S.L., Wu C.H. (2023). Biological and clinical significance of the AGE-RAGE axis in the aggressiveness and prognosis of prostate cancer. J. Food Drug Anal..

[23] Gabrielle P.H. (2022). Lipid metabolism and retinal diseases. Acta Ophthalmol..

[24] Yan Y., Wu Y., Zhao Y., Yang Y., An G., Liu Z., Qi D. (2025). A review on eye diseases induced by blue light: Pathology, model, active ingredients and mechanisms. Front. Pharmacol..

[25] Cougnard-Gregoire A., Merle B.M.J., Aslam T., Seddon J.M., Aknin I., Klaver C.C.W., Garhöfer G., Layana A.G., Minnella A.M., Silva R. (2023). Blue Light Exposure: Ocular Hazards and Prevention-A Narrative Review. Ophthalmol. Ther..

[26] Shang Y.M., Wang G.S., Sliney D.H., Yang C.H., Lee L.L. (2017). Light-emitting-diode induced retinal damage and its wavelength dependency in vivo. Int. J. Ophthalmol..

[27] Hu Z., Zhang Y., Wang J., Mao P., Lv X., Yuan S., Huang Z., Ding Y., Xie P., Liu Q. (2016). Knockout of Ccr2 alleviates photoreceptor cell death in rodent retina exposed to chronic blue light. Cell Death Dis..

[28] Tosini G., Ferguson I., Tsubota K. (2016). Effects of blue light on the circadian system and eye physiology. Mol. Vis..

[29] Daugaard S., Markvart J., Bonde J.P., Christoffersen J., Garde A.H., Hansen Å.M., Schlünssen V., Vestergaard J.M., Vistisen H.T., Kolstad H.A. (2019). Light exposure during days with night, outdoor, and indoor work. Ann. Work Expo. Health.

[30] Contín M.A., Arietti M.M., Benedetto M.M., Bussi C., Guido M.E. (2013). Photoreceptor damage induced by low-intensity light: Model of retinal degeneration in mammals. Mol. Vis..

[31] Wei Q., Liang X., Peng Y., Yu D., Zhang R., Jin H., Fan J., Cai W., Ren C., Yu J. (2018). 17β-estradiol ameliorates oxidative stress and blue light-emitting diode-induced retinal degeneration by decreasing apoptosis and enhancing autophagy. Drug Des. Dev. Ther..

[32] Dow C., Mancini F., Rajaobelina K., Boutron-Ruault M.-C., Balkau B., Bonnet F., Fagherazzi G. (2018). Diet and risk of diabetic retinopathy: A systematic review. Eur. J. Epidemiol..

[33] Orhan C., Er B., Deeh P.B.D., Bilgic A.A., Ojalvo S.P., Komorowski J.R., Sahin K. (2021). Different Sources of Dietary Magnesium Supplementation Reduces Oxidative Stress by Regulation Nrf2 and NF-κB Signaling Pathways in High-Fat Diet Rats. Biol. Trace Elem. Res..

[34] Albouery M., Buteau B., Grégoire S., Martine L., Gambert S., Bron A.M., Acar N., Chassaing B., Bringer M.A. (2020). Impact of a high-fat diet on the fatty acid composition of the retina. Exp. Eye Res..

[35] Keeling E., Lynn S.A., Koh Y.M., Scott J.A., Kendall A., Gatherer M., Page A., Cagampang F.R., Lotery A.J., Ratnayaka J.A. (2022). A High Fat “Western-style” Diet Induces AMD-Like Features in Wildtype Mice. Mol. Nutr. Food Res..

[36] Chang R.C.-A., Shi L., Huang C.C.-Y., Kim A.J., Ko M.L., Zhou B., Ko G.Y.-P. (2015). High-fat diet–induced retinal dysfunction. Investig. Ophthalmol. Vis. Sci..

[37] Hernandez-Castillo C., Shuck S.C. (2021). Diet and Obesity-Induced Methylglyoxal Production and Links to Metabolic Disease. Chem. Res. Toxicol..

[38] Mastrocola R., Nigro D., Chiazza F., Medana C., Dal Bello F., Boccuzzi G., Collino M., Aragno M. (2016). Fructose-derived advanced glycation end-products drive lipogenesis and skeletal muscle reprogramming via SREBP-1c dysregulation in mice. Free Radic. Biol. Med..

[39] Rowan S., Jiang S., Korem T., Szymanski J., Chang M.L., Szelog J., Cassalman C., Dasuri K., McGuire C., Nagai R. (2017). Involvement of a gut-retina axis in protection against dietary glycemia-induced age-related macular degeneration. Proc. Natl. Acad. Sci. USA.

[40] Pan C., Banerjee K., Lehmann G.L., Almeida D., Hajjar K.A., Benedicto I., Jiang Z., Radu R.A., Thompson D.H., Rodriguez-Boulan E. (2021). Lipofuscin causes atypical necroptosis through lysosomal membrane permeabilization. Proc. Natl. Acad. Sci. USA.

[41] O’Leary F., Campbell M. (2023). The blood-retina barrier in health and disease. FEBS J..

[42] Wei L., Mo W., Lan S., Yang H., Huang Z., Liang X., Li L., Xian J., Xie X., Qin Y. (2022). GLP-1 RA Improves Diabetic Retinopathy by Protecting the Blood-Retinal Barrier through GLP-1R-ROCK-p-MLC Signaling Pathway. J. Diabetes Res..

[43] Chan Y.J., Hsiao G., Wan W.N., Yang T.M., Tsai C.H., Kang J.J., Lee Y.C., Fang T.C., Cheng Y.W., Li C.H. (2023). Blue light exposure collapses the inner blood-retinal barrier by accelerating endothelial CLDN5 degradation through the disturbance of GNAZ and the activation of ADAM17. Fluids Barriers CNS.

[44] Rajagopal R., Bligard G.W., Zhang S., Yin L., Lukasiewicz P., Semenkovich C.F. (2016). Functional Deficits Precede Structural Lesions in Mice with High-Fat Diet-Induced Diabetic Retinopathy. Diabetes.

